# Rivaroxaban Failure Presenting With Renal Infarction in a Patient With Persistent Atrial Fibrillation: A Case Report

**DOI:** 10.7759/cureus.52332

**Published:** 2024-01-15

**Authors:** Shudipan Chakraborty, Fnu Salman, Moaaz Baghal, Ayesha Tahir, Abdul Baqi, Hemindermeet Singh, Mohammed Taleb

**Affiliations:** 1 Internal Medicine, Mercy Health - St. Vincent Medical Center, Toledo, USA; 2 Cardiology, Mercy Health - St. Vincent Medical Center, Toledo, USA; 3 Interventional Cardiology, Mercy Health - St. Vincent Medical Center, Detroit, USA

**Keywords:** mitral valve, echocardiography - heart failure - valvular heart disease, echocardiography, left atrial thrombus, thrombous

## Abstract

Direct oral anticoagulants (DOAC) are the preferred choice of anticoagulation for patients with atrial fibrillation. DOACs are always preferred over vitamin K antagonists due to their better safety profile in terms of life-threatening bleeding and decreased need for INR (international normalised ratio) monitoring. Although the most commonly used anticoagulation, failure to DOAC has been reported. Here we present a rare case of rivaroxaban failure presenting with left renal infarction in a patient who had dense spontaneous echocardiographic contrast in the left atrium visualised by transthoracic echocardiography.

## Introduction

DOACs are the first-line anticoagulants in patients requiring long-term anticoagulation for atrial fibrillation. Rivaroxaban acts as a direct Factor Xa inhibitor. Factor Xa is placed just above the thrombin, at the convergence point between extrinsic and intrinsic coagulation pathways. Factor Xa inhibition leads to the prevention of amplified thrombin generation as a single molecule of factor Xa will convert 1000 molecules of prothrombin to thrombin [1]. DOACs have pretty significant advantages over heparin and warfarin which include significantly lower bleeding risk, fewer risk of bone fractures, less need for laboratory monitoring, and preferable pharmacokinetics. For initiating anticoagulation we typically use the CHADSVASc score. For a patient with a CHADSVASc score more than or equal to 2 in males or more than or equal to 3 in females, the recommendation is to do chronic anticoagulation. For a score of 1 in males and 2 in females, the total documented burden of atrial fibrillation influences the decision-making. Age 65-74 is a stronger risk factor than the other factor conferring one CHADSVASc score point [2]. Ischemic stroke due to embolization of the thrombus originating from the left atrium is the most common thromboembolic manifestation of atrial fibrillation; embolization to other locations in the systemic circulation is also rarely found.

## Case presentation

**Figure 1:**
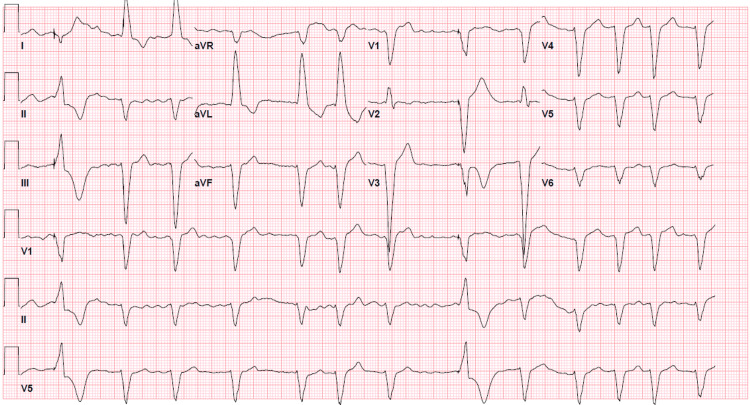
EKG showing atrial fibrillation with occasional ventricular paced complexes

This 60-year-old female patient with a past medical history significant for persistent atrial fibrillation compliant with rivaroxaban, sick sinus syndrome status post permanent pacemaker placement, chronic left bundle branch block (LBBB), non-ischemic cardiomyopathy with an ejection fraction of 35-40% presented to the ED with the chief complaint of shortness of breath and found to be in acute hypoxic respiratory failure requiring 2-3 liter nasal cannula oxygen. The patient was saturating 94% on 2-3 liter nasal cannula oxygen. Hemodynamically, she was stable with a blood pressure of 138/88 mmHg and a heart rate of 75 beats/min. She was started on breathing treatment and intravenous methylprednisolone for the management of chronic obstructive pulmonary disease. An electrocardiogram in the ED showed atrial fibrillation with occasional ventricular paced complexes with premature aberrantly conducted complexes and chronic LBBB (Figure 1). Last echocardiography showed an ejection fraction of 35-40%, pacer wire in the right ventricle, and Grade 1 diastolic dysfunction. Lateral E’ was 6.20 cm/s and Medial E’ was 5.77 cm/s. The last cardiac catheterization showed mild nonobstructive coronary artery disease involving the right coronary artery showing 30% stenosis. Of note, the patient got synchronized cardioversion fairly recently for persistent atrial fibrillation and was compliant with rivaroxaban since the CHA2DS2VASc score was 3. The patient's home medication included rivaroxaban 20 mg daily, metoprolol extended release 100 mg once daily, spironolactone 25 mg once daily, atorvastatin 40 mg once daily, and lisinopril 2.5 mg once daily. Physical examination revealed crackles over bilateral lower lung fields. The rest of the physical examinations were unremarkable. The patient was taking rivaroxaban at least 7 months before this hospitalization.

**Figure 2:**
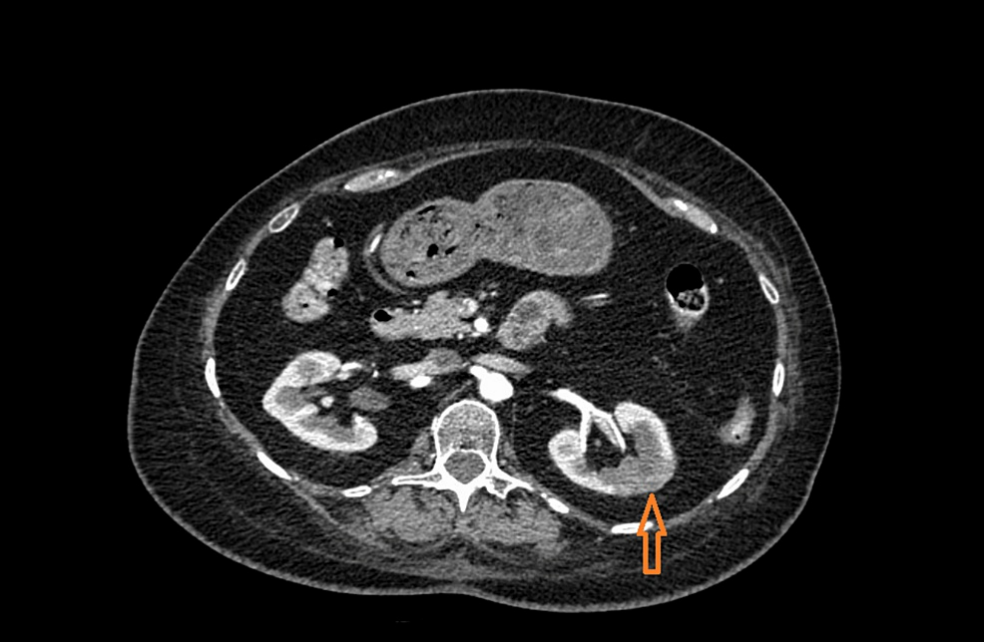
CT of the abdomen and pelvis with intravenous contrast The orange arrow shows left renal infarction.

ED evaluation revealed that the patient was requiring 2 liters of oxygen, and venous blood gas analysis revealed pH 7.318, PCO2 (partial pressure of cabon dioxide) 55.1 mmHg, PO2 (partial pressure of oxygen) 47.1 mmHg, bicarbonate 27.4 mmol/L. High-sensitivity troponins were negative on subsequent checks. The first set of high-sensitive troponin was 20 ng/L which further downtrended to 19 ng/L. Lactate was elevated to 4.3 mmol/L. Subsequent lactate levels were trending up. Urinalysis was unremarkable. Due to persistent lactic acidemia and nonspecific epigastric pain a CT of the abdomen and pelvis with intravenous contrast was obtained which revealed heterogeneous left renal enhancement at the upper pole of the interpolar region of the left kidney highly suggestive of renal infarction (Figure 2).

**Figure 3:**
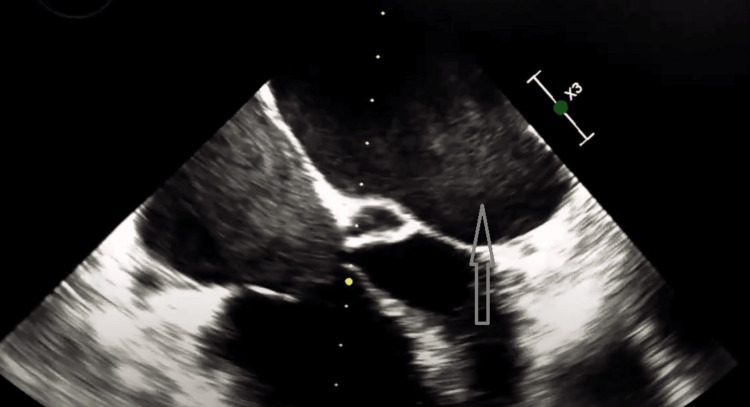
Transthoracic echocardiography Gray arrow showing dense spontaneous echocardiographic contrast in the left atrium.

Urology and vascular surgery were consulted in the hospital and no surgical procedure was planned. Hematology was also consulted and the recommendation was to switch the patient to warfarin and stop DOAC. Transthoracic echocardiography was done and showed dense spontaneous echocardiographic contrast in the left atrium (Figure 3). Transesophageal echocardiography (TEE) was ordered but the patient declined the procedure.

## Discussion

Rivaroxaban is a pretty effective anticoagulation for preventing thromboembolism in patients with atrial fibrillation. However, failure of rivaroxaban is rare but can happen. Some of the predictors of these anticoagulation failures include TEE evidence of dense spontaneous echo contrast and low atrial appendage ejection velocity. Despite oral anticoagulation, people with atrial fibrillation and dense spontaneous echo contrast have a higher chance of cerebral embolism than patients without dense spontaneous echo contrast [3].

TEE-detected thrombi, if present before the initiation of DOAC, can also lead to anticoagulation failure. The development of thrombi can be related to the risk factor which can be stratified from the CHADSVASc score system. Noncompliance is also a reason for the failure of anticoagulation. But in the case of our patient, noncompliance was not an issue and the patient was taking rivaroxaban regularly.

Dense spontaneous echo contrasts are also called “Smoke”. It is defined as the echogenicity of blood in the absence of injected contrast agents. It is an echogenic swirling pattern of blood flow visible on either transthoracic or transesophageal echocardiogram. It is due to the interaction of RBC and plasma protein, especially, fibrinogen [4]. Spontaneous echo contrast is most commonly seen in the left atrium and left atrial appendage on transesophageal echocardiography. Thrombus formation in the left atrial cavity is also associated with mitral valve disease and in those cases smoke-like dense spontaneous echocardiographic contrast would be present [5]. Spontaneous echo contrast is seen in atrial fibrillation, mitral stenosis, mitral valve prosthesis, and left atrial enlargement. All of the above-mentioned conditions will have reduced blood flow velocity, spontaneous echo contrast is also reported in the left ventricle, right atrium, and descending aorta.

## Conclusions

Our case report describes that dense spontaneous echocardiographic contrast is associated with an increased risk of thromboembolism; in this case, renal infarction even in patients who are compliant with anticoagulation. However, a very limited number of studies have been published to correlate the quantitative relationship between spontaneous echo contrast and the risk of thromboembolism. So, large-scale studies are necessary to formulate a correlation between spontaneous echocardiographic contrast and the risk of thromboembolism even with anticoagulation.
